# Epidural Blood Patch in Patients With Hematologic Malignancies: A Case Series

**DOI:** 10.7759/cureus.90906

**Published:** 2025-08-24

**Authors:** Ilaria Vittoria De Martini, Samuel N Rogers, Laurel Truscott, Hasan Ozgur

**Affiliations:** 1 Radiology and Imaging Science, University of Arizona, Banner University Medical Center, Tucson, USA; 2 Pediatrics, University of Arizona, Banner University Medical Center, Tucson, USA

**Keywords:** epidural blood patch, hematologic malignancies, lumbar puncture, lumbar puncture needle, post-dural puncture headache

## Abstract

Background and purpose: Post-dural puncture headaches (PDPH) are caused by cerebrospinal fluid (CSF) leaks that occur after a lumbar puncture (LP). Epidural blood patch (EBP) is an effective treatment option for patients with PDPH in whom conservative treatment fails. Special consideration has to be taken if the patient is affected by any hematological malignancy, due to the fact that EBP carries a theoretical risk of seeding the malignancy into the neuroaxis. The frequent occurrence of immunocompromised status and thrombocytopenia in this patient population warrants individual assessment prior to decision-making. The purpose of our study was to demonstrate that EBP is a safe treatment option if used in the right clinical setting.

Materials and methods: In our study, we report the imaging and clinical findings of five patients with hematologic malignancies who developed PDPH following LP. All patients failed to respond to conservative treatment and required EBP.

Results: The EBP was successfully performed in all five patients. The preprocedural brain and spine MRI findings were concerning for CSF leak, demonstrating subdural fluid collections, pachymeningeal enhancement, and brain sagging. All patients responded favorably to EBP with the resolution of symptoms. In the long-term follow-up, none of them developed postprocedural infections or neuroaxis tumor seeding. Thrombocytopenia did not result in failure of the procedure or any bleeding complications.

Conclusion: EBP is effective in treating conservative therapy-refractory PDPH in a patient population with underlying hematologic malignancies. Due to the increased risks of infection in the setting of immunosuppression, compromised coagulation status related to underlying malignancy, and possible tumor seeding to the neuroaxis, the procedure and possible associated risks should be discussed thoroughly with the patient and family.

Clinical significance: We noticed in our practice that there is a lack of deep understanding of a specific patient population affected by hematologic malignancies developing postdural puncture headaches after an LP. As of today, there are no well-established guidelines regarding this specific situation, and every proceduralist has to make a decision whether they feel comfortable performing an EBP when conservative treatment fails, with the possible risk of disease spreading, infection, and bleeding. With our paper, we offered insights into our practice and results, and hope to serve as a helpful resource for proceduralists who are facing a similar clinical setting.

## Introduction

Post-dural puncture headaches (PDPH) are postural headaches caused by low intracranial pressure secondary to cerebrospinal fluid (CSF) leaks that occur hours to days after a lumbar puncture (LP) or inadvertent dural puncture during procedures such as epidural steroid injections or epidural anesthesia; they are encountered in up to 32% of the patients undergoing LP, and the incidence of PDPH is higher with larger needles (such as 16 gauge or 17 gauge) with conventional needle tips (such as the Tuohy needle). In most cases, the headache is self-limited and resolves spontaneously or with conservative medical treatment [1]. The pathophysiology of these headaches is poorly understood. The widely accepted explanation for PDPH is a continuous leakage of CSF through the needle tract, resulting in decreased CSF pressure and volume, thus resulting in tension on the meninges and pain in sensitive brain structures [2,3]. Another proposed mechanism for PDPH is vasodilatation and increased brain volume secondary to compensatory activation of cerebral adenosine receptors [4].

The headaches are positional in nature, improving in the recumbent position and worsening when sitting or standing. Photophobia, neck stiffness, nausea, vomiting, dizziness, and tinnitus may be associated with PDPH [5].

Medical treatment for PDPH is rest, hydration (oral or intravenous), oral caffeine, and intravenous analgesics [6]. In patients with no or limited response to conservative treatment, EBP offers a safe and effective treatment option with a reported success rate of 70-98%. Autologous blood is routinely used for this procedure, which is usually obtained from the patient’s upper extremity vein or from a central venous catheter using sterile technique [7].

Patients with hematologic malignancies often undergo LPs for diagnostic and therapeutic purposes, such as to administer intrathecal (IT) chemotherapy [8,9]. Treatment of PDPH in this patient population is surrounded by uncertainty because of the unique challenges of safely performing EBP in these patients. First, EBP carries a theoretical risk of seeding malignancy into the epidural space and possibly into the neuroaxis if malignant cells are present in the autologous blood sample [10]. Second, these patients are often immunocompromised, making them more vulnerable to infections [11], thus making a sterile technique even more crucial during the procedure [12]. Third, these patients are often thrombocytopenic [13]. With decreased platelets, the coagulation occurs at a slower rate [14] and may potentially decrease the success rate of EBP.

Prior to EBP, other possible causes of headaches, such as intracranial hemorrhage, meningitis, sinus thrombosis, or spread of the malignancy to the brain, must be excluded [7]. If the patient has a typical clinical presentation, such as positional headaches starting hours to several days after the LP, lack of focal or lateralizing findings on neurological examination, and no significant change in the baseline mental status, further imaging to exclude other etiologies is usually not indicated. EBP can be performed in these patients if there are no contraindications [7]. 

Currently, there are no established guidelines or recommendations that thoroughly address these challenges regarding EBP in patients with hematologic malignancies and PDPH. The provider performing EBP should be experienced with epidural techniques and should use smaller, pencil-point needles to avoid an inadvertent dural puncture. A recent multisociety consensus report in 2023 was limited on this topic secondary to sparse literature and case reports [15]. In this paper, we add to the literature by presenting our experience with five patients with hematologic malignancy who were safely and effectively treated with EBP for PDPH.

## Materials and methods

Patient population

The study was conducted at the University of Arizona, Banner University Medical Center. This single-center, retrospective study was approved by the local ethics committee (protocol number STUDY00004001, approved on 24/6/2024). The written consent requirement was waived. Our radiology information system was searched for patients undergoing EBP in the setting of PDPH between November 2013 and November 2023. Forty-five patients underwent EBP. Six of the 45 patients who underwent EBP presented after a previously performed diagnostic/therapeutic LP in the setting of an underlying hematologic disease. One patient had to be excluded because the hematologic diagnosis could not be confirmed. As per protocol in our institution, all patients were assessed by a pediatric or adult hematologist/oncologist for their PDPH. Neurology consultation was obtained when deemed indicated. Additionally, prior to the EBP, all patients were evaluated by a neuroradiologist to confirm that the patients’ headaches were compatible with PDPH. Onset hours up to several days after the LP, in conjunction with positional nature (headaches getting significantly better in a supine or recumbent position and worsening in a seated or upright position), were considered compatible with PDPH. The presence of signs concerning meningitis was also excluded prior to performing the EBP. All of the patients were treated conservatively for at least eight days before the EBP. Peripheral blood smear was performed in three patients in order to rule out any circulating blast cells; we were unable to confirm whether or not the other two patients had a peripheral blood smear performed prior to their EBP, as the electronic medical record system was different at the time in our institution. The EBP procedure was extensively discussed with the patients, their families, and the referring oncologist in three patients. All patients had a contrast-enhanced MRI of the brain, and two patients had an MRI of the lumbar spine before the EBP. 

Clinical data analysis 

We retrospectively reviewed the medical charts and imaging findings of the five patients with hematologic malignancies and refractory PDPH included in the study. Age, sex, underlying hematologic disease, hypertension, diabetes, platelet count, and hematocrit level, as well as the presence of other comorbidities (Table 1), and, if available, MRI results, were recorded.

**Table 1:** Summary of patient demographics, comorbidities, conservative treatment duration, and relevant preprocedural laboratory values Hgb: hemoglobin; INR: international normalized ratio; ALL: acute lymphoblastic leukemia; LP: lumbar puncture

Patient No.	Age	Gender	Malignancy	Number of LP before EBP	Conservative treatment duration	Comorbidities	Hgb value	Hgb post-transfusion	INR	Platelets	Platelets post-transfusion
1	16	M	Burkitt lymphoma	4	12 days	None	8		1.1	191	
2	19	F	B cell ALL	3	8 days	None	7.9		1	25	50
3	21	F	B cell ALL	3	14 days	Diabetes	4.4	8.9	0.9	9	32
4	47	M	Burkitt lymphoma	1	50 days	Chronic kidney disease	11.2		1.1	129	
5	29	F	B cell ALL	8	7 days	Chronic kidney disease	9.9		1	75	

Procedure protocol

All the procedures were performed in an interventional radiology suite with digital subtraction angiography equipment by an attending neuroradiologist or a neuroradiology fellow under the direct supervision of a neuroradiology attending. A 22-gauge (G) Quincke-type or a 22-G Tuohy or 16-G Tuohy spinal needle, based on the preference of the proceduralist, was used to gain access to the posterior epidural space at the same level as the previously performed LP (Table 2). Autologous blood was used for all the procedures.

**Table 2:** Summary of the epidural blood patch technique across patients and clinical outcomes EBP: epidural blood patch; LP: lumbar puncture

Patient No.	Blood volume	Improvement timeframe	Result of epidural blood patch	Number of LP after EBP	Recurrent headache	Needle	CNS disease-free follow-up on record
1	15 ml	24 hours	Complete relief in 48 hours	1	No	22 G Quincke	16 months
2	15 ml	72 hours	Complete relief in 60 hours	1	No	22 G Quincke	10 months
3	15 ml	24 hours	Complete relief in 24 hours	1	No	22 G Quincke	26 months
4	5 ml	Unavailable	Significantly improved after 7 days	3	No	16 G Tuohy	57 months
5	25 ml	Unavailable	Significantly improved at 22 days	1	No	20 G Tuohy	59 months

MRI analysis

Brain MRI findings were reviewed with specific attention to pachymeningeal thickening and enhancement, subdural fluid collections, subdural hemorrhage, decreased mamillopontine distance, brainstem caudal tonsillar displacement, pituitary enlargement, and venous engorgement. A mamillopontine distance less than 5.5 mm was considered abnormal [16].

Lumbar MRI findings were reviewed with specific attention to extradural fluid collections (the presence of fluid intensity outside the dura is considered a spinal longitudinal extradural fluid collection) (Table 3).

**Table 3:** Summary of MRI imaging findings of all patients Sagging of the brain is considered when the mamillopontine distance is less than 5.5 mm or cerebellar tonsils are greater than 5 mm below the foramen magnum. N/A: not applicable; SLEC: spinal longitudinal extradural fluid collection; EBP: epidural blood patch

Patient No.	Dural thickening	Dural enhancement	Subdural fluid/blood	Sagging brain	Venous engorgement	Pituitary hyperemia/ enlargement	SLEC	Days from brain MRI to EBP
1	Yes	Yes	Yes	Yes	Yes	Yes	Yes	5
2	No	No	Yes	No	No	No	Yes	6
3	Yes	Yes	Yes	Yes	Yes	Yes	N/A	2
4	Yes	Yes	No	Yes	No	No	N/A	9
5	Yes	Yes	Yes	Yes	Yes	Yes	N/A	3

## Results

**Figure 1:**
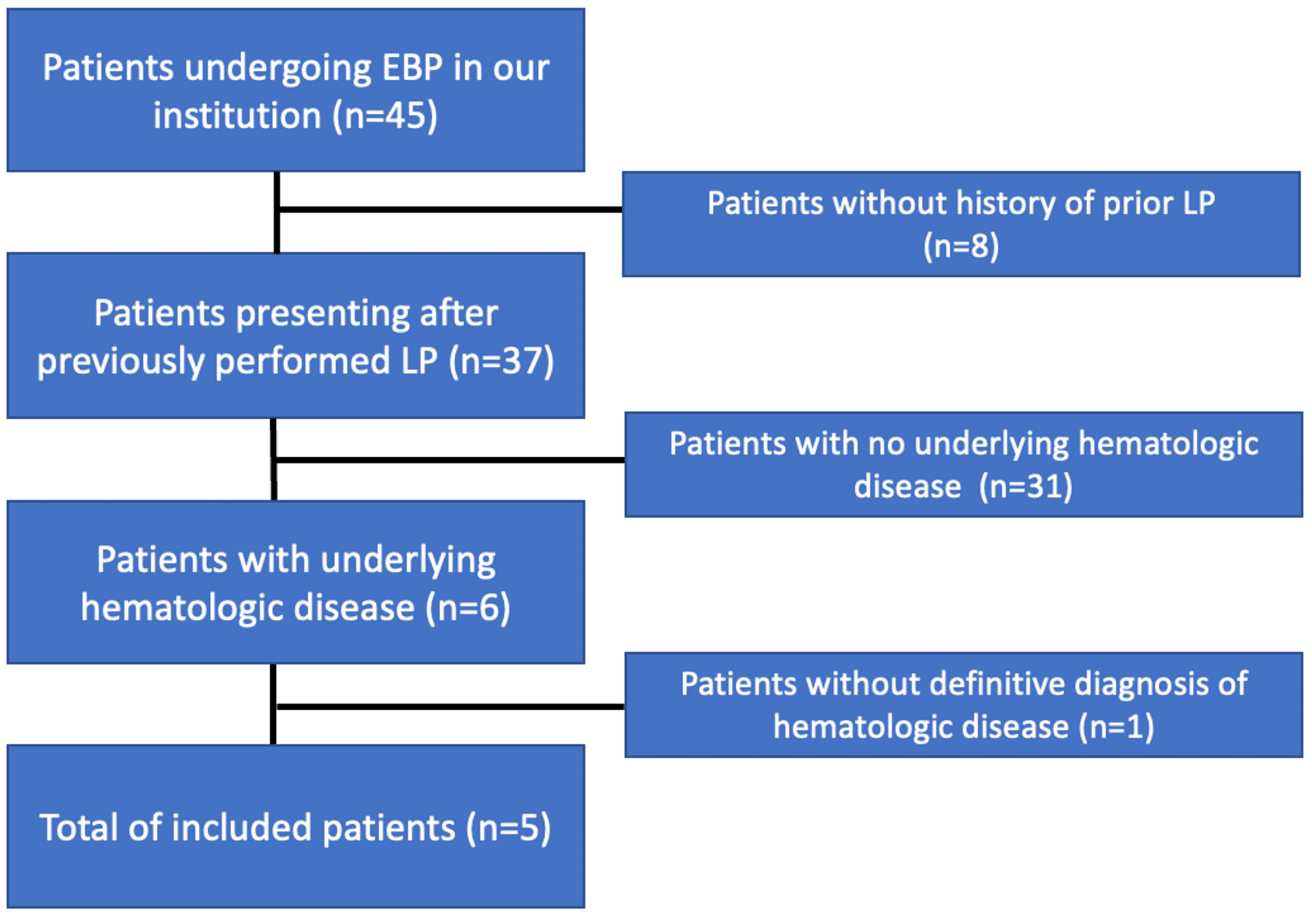
Study flowchart EBP: epidural blood patch; LP: lumbar puncture

A total of 45 patients underwent EBP. Six of these had previously received a diagnostic or therapeutic LP for an underlying hematologic condition. One of these six was excluded due to an unconfirmed hematologic diagnosis. Five patients (age range 16-47 years old, mean age 26.4 years, three females, two males) were included in this study (Figure 1).

Of the five patients with hematologic malignancies undergoing EBP, two patients had the underlying diagnosis of Burkitt’s lymphoma, and three patients had acute lymphocytic leukemia (ALL). None of the patients had reported neuroaxis involvement at the time of EBP. All five patients presented with positional headaches following an LP. Two patients complained additionally of symptoms of nausea, one patient of intermittent neck stiffness, and one patient suffered from double vision. 

All five patients underwent a trial of conservative treatment for a minimum of eight days prior to EBP. Conservative treatment included rest, hydration, caffeine, oxycodone, Tylenol, Benadryl, magnesium, riboflavin, ibuprofen, Fioricet, lidocaine patch, and alternating ice and heat packs, as well as morphine. 

The preprocedural platelet count ranged between 32,000/mcL and 129,000/mcL. Two patients presented initially with a platelet count of 9,000/mcL and 25,000/mcL and thus received platelet transfusions immediately prior to the EBP. Their platelet levels increased to 32,000/mcL and 50,000/mcL, respectively.

Three patients’ peripheral blood smears were reviewed up to 72 hours before the EBP. The results were negative in all three of these patients. We were not able to find any peripheral blood smear or flow cytometry results immediately prior to the EBP in the remaining two patients in the available electronic medical records. 

All patients reported complete or near-complete resolution of their headaches, mostly between 24 and 72 hours following the EBP. Patient 4 and Patient 5 had a follow-up appointment with the provider after seven and 22 days, respectively, and reported significant improvement of their symptoms. The patients had additional clinical follow-up between 10 months and 59 months. None of the patients had documented seeding of their malignancy in the epidural space or at the injection site, or elsewhere in the neuroaxis in the clinical follow-up. None of the patients experienced infection or bleeding related to the EBP. There were no reported postprocedural complications. 

MRI findings

All five patients had a preprocedural brain MRI to rule out other possible causes of headaches. The studies were performed two to nine days prior to the EBP (Patient 1: five days, Patient 2: six days, Patient 3: two days, Patient 4: nine days, Patient 5: three days).

**Figure 2:**
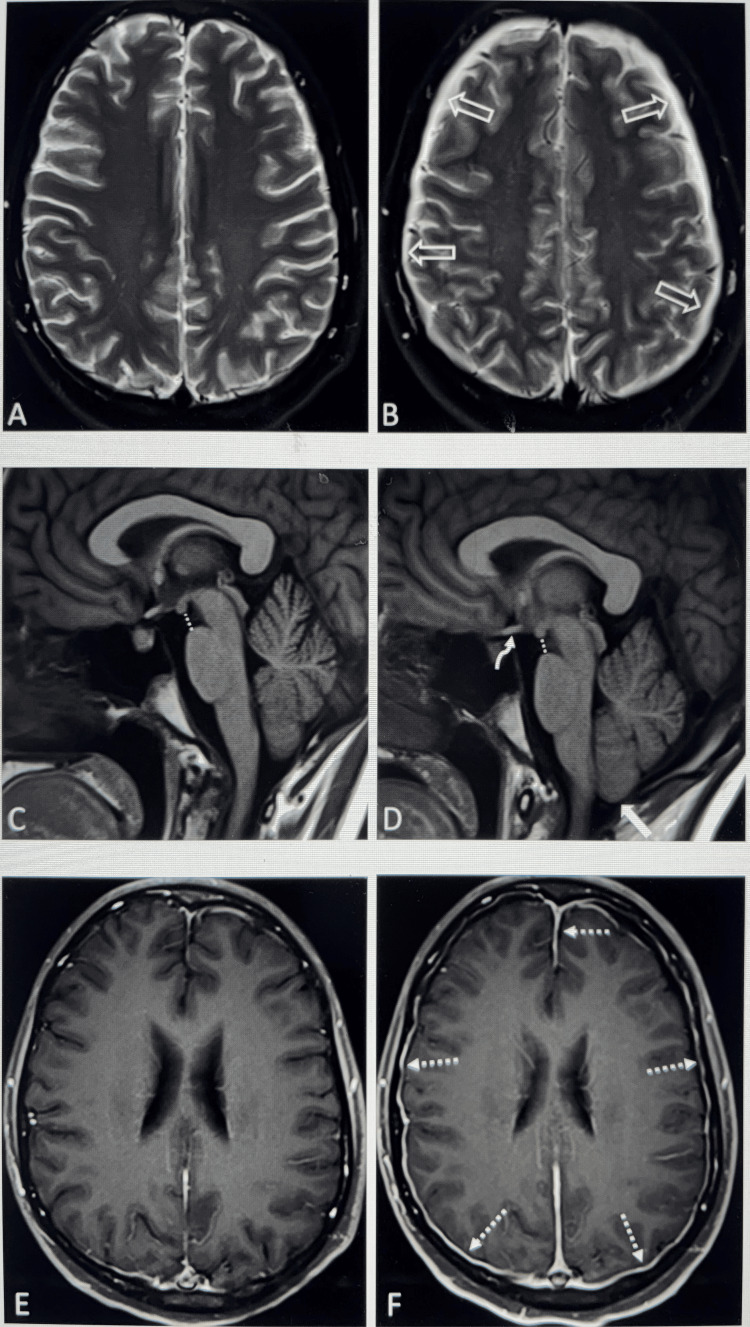
Brain MRI (Patient 1) Patient 1 with successive brain MRIs after repeated lumbar punctures and worsening post-dural puncture headaches, showing new intracranial findings suggesting intracranial hypotension and CSF leak. Patient 1 had four LPs before EBP. After the second lumbar puncture, an MRI was obtained for a mild to moderate headache (A, C, E), showing a normal T2-weighted image (A). After two more lumbar punctures, an MRI was repeated 10 days after the first MRI due to worsening headaches (B, D, F), with new bilateral subdural fluid collections (B, open white arrows). Baseline sagittal T1-weighted images show normal position of the cerebellar tonsils and normal mamillopontine distance (C, dotted line). Follow-up sagittal T1 (D) shows subtle narrowing of the mamillopontine distance (dotted line), inferiorly positioned cerebellar tonsils (solid arrow), and drooping optic chiasm (curved arrow). Baseline axial post-contrast T1 turbo spin echo images (E) show no abnormal enhancement, with abnormal pachymeningeal enhancement bilaterally on the follow-up 10 days after (F, dotted arrows). CSF: cerebrospinal fluid; LP: lumbar puncture; EBP: epidural blood patch

Patients 1, 3, 4, and 5 demonstrated similar intracranial findings: there was evidence of pachymeningeal thickening and enhancement in the T1 postcontrast sequence, hyperintense thin bilateral subdural fluid collections, and venous engorgement in the fluid-attenuated inversion recovery (FLAIR) sequence without mass effect (Figure 2). 

Additionally, in all patients, there was evidence of brain sagging, best demonstrated in the T1 sagittal sequence (Figure 2D). 

**Figure 3:**
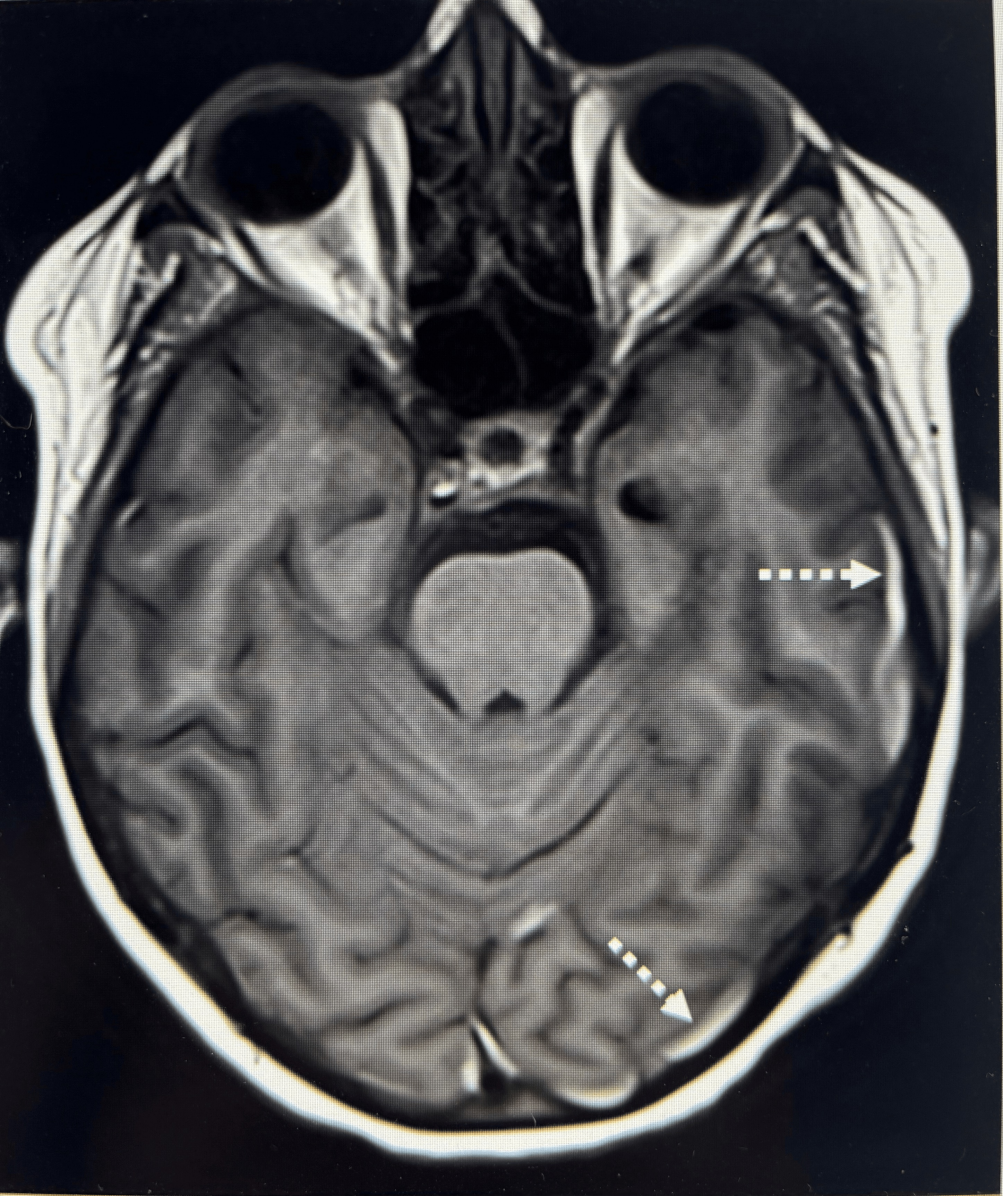
Brain MRI (Patient 2) Brain MRI of Patient 2, performed before EBP. Axial unenhanced T1-weighted sequence reveals subdural hemorrhage shown by thin, hyperintense subdural fluid collections in the left temporal and occipital regions (dashed arrows). EBP: epidural blood patch

Patient 2 demonstrated slightly atypical findings: the brain MRI performed six days before the EBP procedure demonstrated a small amount of layering intradural/subdural hemorrhage in the axial susceptibility-weighted angiography (SWAN) and T1 sequence; however, no FLAIR hyperintense subdural fluid collection was seen (Figure 3).

**Figure 4:**
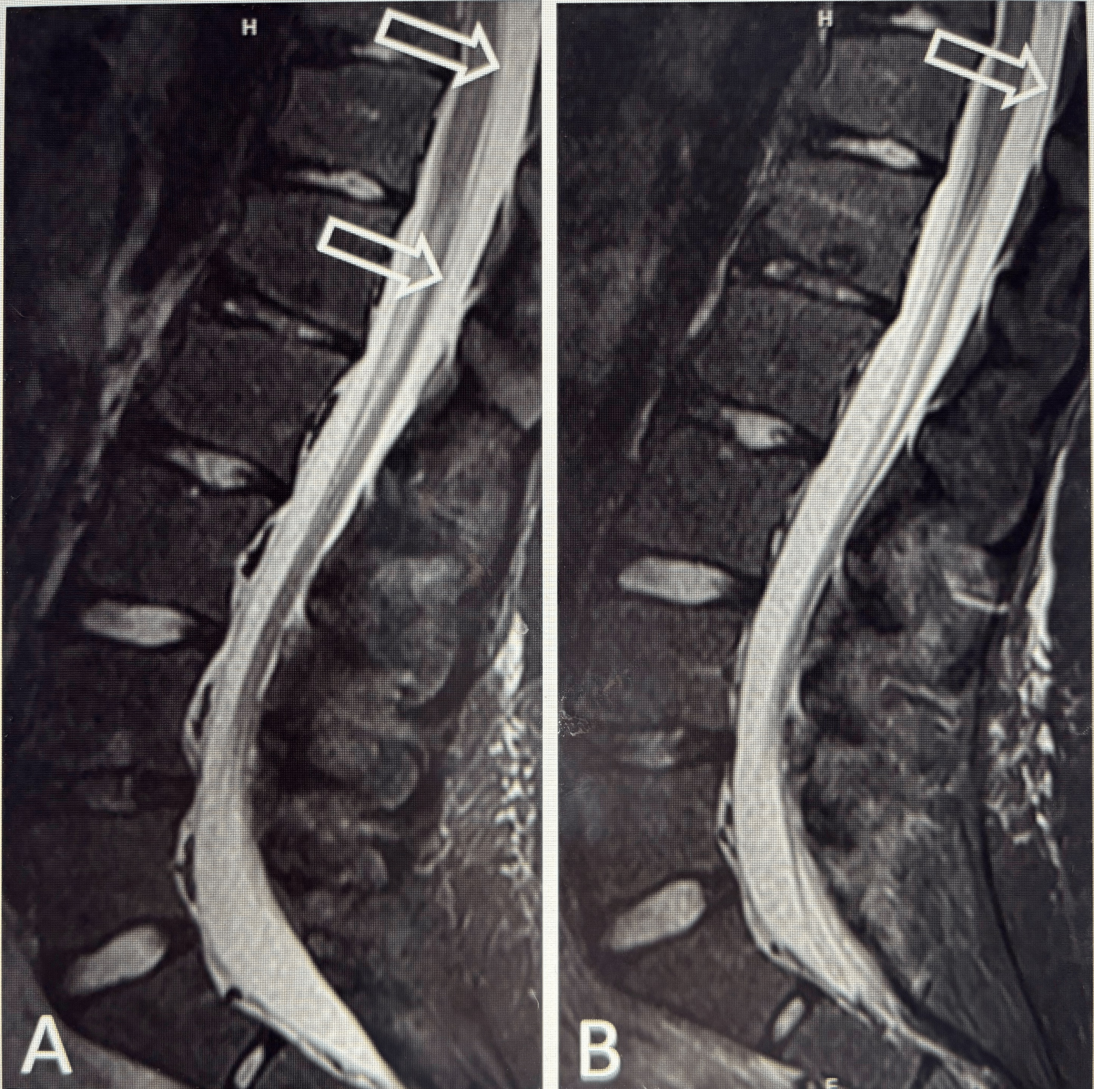
Lumbar spine MRI (Patient 1) Epidural fluid collection on the lumbar spine MRI of Patient 1, which nearly completely resolved after EBP. The sagittal STIR image of the lumbar spine four days before the EBP shows a large fluid collection posterior to the dura (A, open arrows), which significantly decreased in the subsequent MRI five days after the EBP (B, open arrow). Note the position of the dura, which is displaced anteriorly close to the conus medullaris in the pre-EBP MRI versus the normal posterior positioning close to the ligamentum flavum in the post-EBP MRI. EBP: epidural blood patch; STIR: short tau inversion recovery

In the postcontrast T1 sequence, there was evidence of trace pachymeningeal thickening. Patients 1 and 2 additionally had preprocedural spine MRIs, which both demonstrated a spinal longitudinal extradural fluid collection (SLEC). This finding was consistent with CSF leak (Figure 4). 

**Figure 5:**
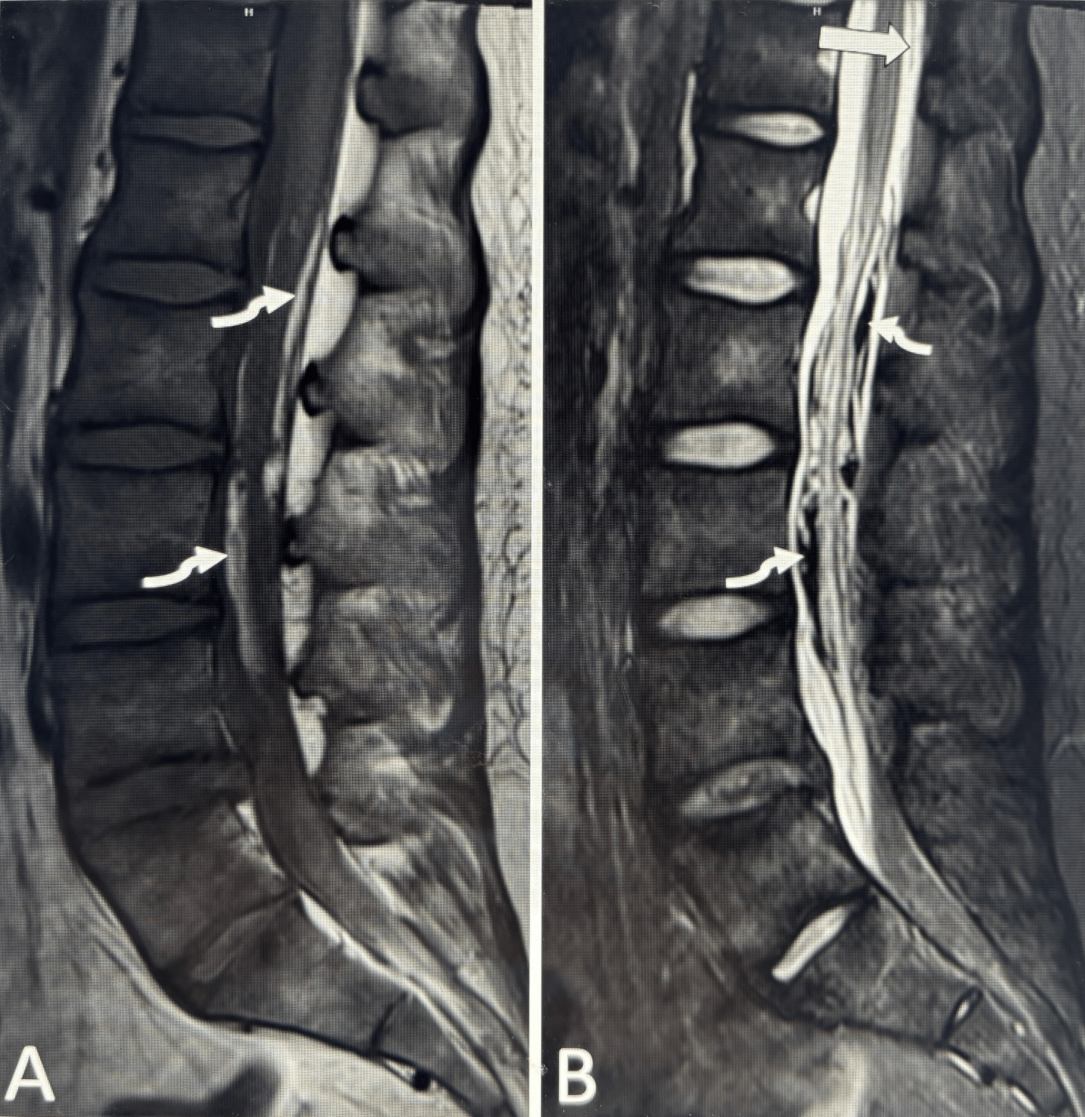
Lumbar spine MRI (Patient 2) Lumbar spine MRI of Patient 2, performed before EBP. Sagittal T1-weighted (A) and sagittal T2-weighted (B) images of the lumbar spine reveal T1 hyperintense and T2 hypointense signals within the thecal sac related to blood products from a recent traumatic LP (curved arrows). There is a thin layer of fluid dorsal to the dura at the T12 level (solid arrow), likely representing epidural fluid from a CSF leak. LP: lumbar puncture; CSF: cerebrospinal fluid

Both spine MRIs demonstrated evidence of epidural thickening and nodularity, and Patient 2 showed additionally increased T1 and decreased T2 signal intensity within the thecal sac with fluid/fluid level, which was consistent with IT blood products (Figure 5).

This finding was thought to be secondary to the LP performed five days prior.

Patients 1, 2, and 5 had a follow-up MRI of the brain after the EBP. Patients 2 and 5 demonstrated complete resolution of the intracranial findings; meanwhile, Patient 1 demonstrated persistent findings of pachymeningeal enhancement and FLAIR hyperintense subdural fluid collection. Patient 1, however, showed improved spinal findings with SLEC resolution. 

## Discussion

In our small case series, we demonstrate both safety and efficacy of EBP in patients with hematologic malignancy and PDPH. In patients with hematologic malignancies, there are no established guidelines regarding EBP for the treatment of PDPH. In the current literature, there are only a few case reports and small case series discussing EBPs performed in patients with leukemia or lymphoma [17-19], which were cited by a recent consensus report on PDPH as evidence that the risk of malignancy seeding is low [15].

When performing an LP, but especially for patients with elevated risk, such as thrombocytopenia, it is advisable to use smaller, pencil-point needles (for example, the 22G Whitacre needle) to minimize the risk of developing PDPH [1].

There are three challenges unique to patients with hematologic malignancies who develop PDPH refractory to conservative treatment and are candidates for EBP. The first challenge is the potential of seeding the hematologic malignancy into the epidural space or other parts of the neuroaxis. The second challenge is the immunocompromised status of many of these patients, making them more vulnerable to procedure-related infections. Finally, the coagulation status of these patients is compromised, often resulting in an increased risk for bleeding complications as well as potentially decreasing the success rate of the procedure due to delayed or incomplete coagulation of the injected blood. 

Among the published series and case reports, there is only one case of potential malignancy seeding related to EBP. One patient with diffuse large B-cell lymphoma developed a new CNS lesion involving the corpus callosum (CC) five months after an EBP. The authors argued that the disease was most likely related to metastasis from the periphery rather than EBP-related seeding, as it is unlikely for the disease to spread from an L2/L3 epidural approach directly to the CC unless there was inadvertent IT injection. Additionally, CC involvement without leptomeningeal disease would be extremely rare even if there was inadvertent dural puncture. Thus, the authors concluded that the new disease was probably not caused by seeding [19]. We were unable to find any additional reports in the literature demonstrating neuraxial seeding of a hematologic malignancy from EBP or any other spinal intervention. It should be noted that patients with hematologic malignancies may undergo neuraxial anesthesia, during which the dura may be punctured or epidural catheters may be inserted [13]. 

Proceduralists who are considering EBP for treatment of PDPH in patients with hematologic malignancy should consider the immune status of these patients due to their underlying malignancy and effects of chemotherapy, which increases the risk of infection [12,13]. The risk is both in the epidural space as well as meningitis if there is inadvertent dural puncture; thus, it is recommended to pay particular attention to using sterile techniques when performing invasive procedures. However, the risk of infection is still rare in this patient population, with most of the epidural infections seen in patients with long-term epidural catheters [20]. Many studies in different patient populations with blood-borne pathogens have shown that the risk of spinal infection related to neuraxial interventions is very low [21].

The hematologic status of the patients should be thoroughly evaluated and screened for the presence of coagulopathy and thrombocytopenia. A low platelet count could potentially decrease the efficacy of the procedure, as it is postulated that the EBP is effective due to coagulation of the injected blood in the epidural space, acting as a plug to stop the CSF leak [3]. Thrombocytopenia, isolated or related to hematologic malignancy, has been discussed previously [4,22]. Howard et al. have retrospectively evaluated 958 children with ALL who underwent 5223 LPs. None of the patients developed bleeding complications, including 29 procedures done with platelet levels of 10,000/mcL or less (150,000 to 400,000 platelets per microliter is considered a normal platelet count) [22]. Ho et al. have searched the Medical Literature Analysis and Retrieval System Online (MEDLINE) database between 1946 and 2017 with the keywords including thrombocytopenia, leukemia, lymphoma, LP, and spinal hematoma. There was no reported spinal hematoma [4]. In our study, the targeted platelet count was at least 25,000/mcL before proceeding to perform the EBP. In two cases, we transfused up to two units of platelets in order to achieve an acceptable platelet count. 

We reviewed additional cases in the literature that confirm the low risk of seeding in patients with hematological malignancies treated with EBP, with no patients in our series affected by malignant seeding. Some author points out the benefits of using flow cytometry of the patient’s blood to screen and exclude the presence of malignant blast cells prior to EBP [18]. Our institutional oncology protocol of peripheral blood smear review proved sufficient in our patient population.

The following study limitations must be acknowledged. First, this retrospective study included only EBP procedures performed in the neuroradiology department of one institution and with a limited sample size. Future studies with larger patient cohorts are required to further validate the results and screen for additional possible complications of the procedure in such a patient population. Second, longer follow-up and continuous disease monitoring are required. Third, although EBP with the patient’s own blood is considered standard treatment in patients with refractory PDPH, the study lacks a control group with a different treatment approach. 

## Conclusions

In the current literature, there are no confirmed cases of seeding of hematologic malignancy after an EBP. We were able to confirm this observation in our study, and furthermore, we demonstrated that the procedure appears to be an effective treatment option for patients with underlying hematologic malignancy who develop PDPH. Due to the fact that this patient population is immunosuppressed and therefore more prone to infection, the procedure and possible associated risks should be discussed thoroughly with the patient and family. Likewise, abnormal hematologic tests, such as low platelets, may potentially decrease the success rate of the procedure and must be addressed accordingly. 
